# Comparison of two formulas for calculating intraocular lens in patients with angle-closure glaucoma

**DOI:** 10.1097/MD.0000000000040387

**Published:** 2024-11-08

**Authors:** Zhen Ji, Jing Ma, Zhonghua Sun, Miaomiao Zhang

**Affiliations:** a Department of Ophthalmology, The Second People’s Hospital of Jinan, Jinan, PR China.

**Keywords:** angle closure, artificial crystal, glaucoma

## Abstract

This study compares the accuracy of SRK/T and Barrett Universal II intraocular lens (IOL) measurement formulas for cataract phacoemulsification combined with intraocular lens implantation in angle-closure glaucoma (ACG) patients. Prospective serial case studies were conducted. A total of 146 patients (146 eyes) with ACG complicated with cataract who underwent cataract surgery with atrial angle closure ≤180° were observed. All the patients were divided into 2 groups according to different intraocular lens calculation formula, with 73 cases in each group (73 eyes). SRK/T intraocular lens calculation formula was used in group A, and Barrett intraocular lens calculation formula was used in group B. The visual acuity, the best corrected visual acuity, intraocular pressure and central anterior chamber depth were significantly improved in 146 eyes 3 months after operation, and the differences were statistically significant. There was statistically significant difference in postoperative spherical equivalent (SE) between the 2 groups (*t* = 2.147, *P* = .033), and there was statistically significant difference in preoperative expected refraction between group A and postoperative SE (*t* = 4.221, *P* < .001). There was no significant difference between preoperative expected diopter and postoperative SE in group B (*t* = 1.857, *P* = .065). The difference in absolute predicted error between the 2 groups was statistically significant (*t* = 4.929, *P* < .001). Axis length was negatively correlated with SRK/T formula and Barrett Universal II formula absolute refractive error value (ME) (group A: *r* = ‐0.740, *P* < .001; group B: *r* = ‐0.532, *P* < .001), there was A significant negative correlation between absolute refractive error value and anterior chamber depth in both groups (group A: *r* = ‐0.382, *P* = .001; group B: *r* = ‐0.358, *P* = .002). The cataract phacoemulsification combined with intraocular lens implantation is safe and effective for ACG patients with cataract. For such patients, the Barrett Universal II intraocular lens measurement formula may be more accurate after surgery.

## 1. Introduction

Primary angle-closure glaucoma is the predominant type of glaucoma in Asia. Previous studies have indicated that ocular risk factors for primary angle closure glaucoma are shallow anterior chamber depth, short axial length, and increased lens thickness. Among these parameters, the lens are considered to play a vital role in the pathogenesis of angle closure glaucoma. The lens factors of angle closure glaucoma mainly include increased lens thickness, forward displacement and pupillary block. Removing the lens through cataract phacoemulsification can deepen the anterior chamber and alleviate angle congestion, pupillary block, and lens factor.

Accurate calculation of intraocular lens (IOL) strength is the key to accurately predict the postoperative refractive state of cataract patients. However, at present, various IOL calculation formulas have different accuracy in predicting postoperative diopter.^[1,2]^ Patients with primary angle closure glaucoma have anatomical characteristics such as short axial length, shallow anterior chamber, and narrow angle of the chamber, and the position of the lens is relatively anterior,^[3]^ resulting in a large deviation of postoperative diopter accuracy. Although there are many research literatures on different calculation formulas of IOL power,^[4–7]^ there are few reports on the prediction accuracy of different IOL calculation formulas for angle-closure glaucoma (ACG) patients undergoing lens surgery.

In order to make the calculation of IOL power more accurate, the fifth generation of IOL power calculation formula appears, which is represented by the Barrett Universal II formula. There is no report on the application of the fifth-generation IOL calculation formula in patients with ACG. At present, the SRK/T IOL calculation formula is still the most commonly used formula in clinical work. In this study, we compared the accuracy of the SRK/T and Barrett Universal II IOL calculation formulas for patients with ACG, and provided a theoretical basis for choosing a more appropriate IOL calculation formula.

## 2. Methods

This is a case series observational study. This study included 146 patients (146 eyes) with ACG, who underwent cataract phacoemulsification combined with IOL implantation in the Second People’s Hospital of Jinan City from December 2019 to July 2020. There were 56 males (56 eyes) and 90 females (90 eyes), aged from 50 to 92 years old, with an average of (68.68 ± 8.31) years old. The patients were divided into 2 groups according to different IOL calculation formula. Patients in group A were treated with SRK/T, and patients in group B were treated with Barrett Universal II. Inclusion criteria: 1. The selected eyes were diagnosed as angle-closure glaucoma; 2. The intraocular pressure (IOP) was >21 mm Hg (1 mm Hg = 0.133 kPa), and the angle of adhesion of the anterior chamber was ≤180°; 3. The lens was opacified, and the uncorrected visual acuity was <0.5. Exclusion criteria: 1. Significant asymmetry in the depth of the anterior chamber of both eyes; 2. Spherical diopter >+2.00 D or <‐6.00 D, cylinder diopter >+1.00 D or <‐1.00 D; 3. History of previous eye surgery and history of other diseases; 4. Obvious systemic or other diseases that may affect relevant examinations. All the selected cases were wedge-shaped opacity and swelling of the lens cortex, and the IOP at first diagnosis ranged from 36 to 63 mm Hg, which could be reduced to <25 mm Hg after the application of local or systemic IOP drugs.

After preoperative IOP control, uncorrected visual acuity, best corrected visual acuity (BCVA), IOP, slit lamp microscopy, fundoscopy, corneal endothelium examination, and ocular B-ultrasound were performed preoperatively. Goldmann applanation tonometer was used for IOP measurement; refraction was checked by computer refractometer RM-100 (Topcon Company, Japan), the average value of 3 times measurements was taken, and spherical lens + 1/2-cylinder lens was recorded as equivalent spherical lens (spherical equivalent, SE).

IOL power measurement: IOL Master was used to measure the axial length and corneal curvature, central anterior chamber depth (ACD), and lens thickness by the same professional. The final result is the average of 10 repeated measurements. According to the axial length and corneal curvature measured by the IOL-Master instrument, 2 formulas were used to calculate the required IOL diopter, and the target diopter after surgery was 0.00–0.50 D.

All operations were performed by the same experienced physician using an Infiniti phacoemulsification machine (Alcon, USA). All surgical procedures were smooth and no intraoperative complications occurred. Thirty minutes before surgery, the pupils were fully dilated with compound tropicamide eye drops, and topical anesthesia was performed with tetracaine eye drops. A clear corneal incision was made at 11:00, lateral incisions were made at 2:00 and 10:00. Subsequently, a continuous annular capsulorhexis of approximately 5.5 mm in diameter was performed. Then water separation, water layering, phacoemulsification of lens nucleus, cortical injection and suction, anterior and posterior capsular polishing, intraocular lens implantation (Alcon Hydrophobic Acrylate Posterior Chamber Intraocular Lens [SN60WF]) in the capsular bag is performed step by step, suction the elastomer, adjust the lens position to center the IOL, and ensure that the posterior capsule is not wrinkled. Finally, a watertight surgical incision keeps the anterior chamber stable. After operation, tobramycin and dexamethasone eye drops (Dianbishu eye drops, Japan Santen) were instilled in the eyes, 4 times a day, decreased once a week, and stopped after 4 weeks. Pralprofen eye drops were taken 4 times a day and discontinued after 1 month.

The observation indicators included uncorrected visual acuity, BCVA, IOP, refractive status, central ACD and absolute refractive error value (the absolute difference between the expected postoperative refractive power and the postoperative equivalent spherical difference [ME]) during the 3-month follow-up.

SPSS 26.0 statistical software (SPSS Corporation, USA) was used for statistical analysis. The number was expressed as mean ± SD. The data in this study were tested by Kolmogorov–Smirnov and were in line with normal distribution. The *t* test of 2 independent samples was used to compare the preoperative and postoperative visual acuity, BCVA, IOP, ACD, and ME. The correlation between Al and Me was analyzed by Pearson correlation. *P* ≤ .05 was considered statistically significant.

## 3. Results

There was no statistically significant difference in preoperative general data between the 2 groups. The axial length was (22.32 ± 0.57) mm in group A and (22.28 ± 0.66) mm in group B, and the difference between the 2 groups was not statistically significant (*t* = 0.392, *P* = .696). Postoperative visual acuity of the 2 groups was significantly improved compared with preoperative visual acuity (group A: *t* = ‐0.47, *P* < .001; group B: *t* = ‐0.36, *P* < .001). The IOP of the 2 groups after operation was significantly lower than that before operation (group A: *t* = 5.869, *P* < .001; group B: *t* = 5.598, *P* < .001). The preoperative ACD of all patients was 2.14 ± 0.26 mm, and the postoperative ACD was 3.12 ± 0.21 mm. The depth of anterior chamber was significantly deeper after surgery than before (*t* = 25.053, *P* < .001) (Table 1).

**Table 1:** Comparison of basic information of 2 groups of IOL implantation (D, mean ± SD).

Groups	Eyes (male/female)	Age (years)	Preoperative IOP (mm Hg)	Degree of IOL (D)	Preoperative vision (LogMAR)
Group A	26/47	68.68 ± 8.34	18.55 ± 7.45	23.23 ± 1.91	0.67 ± 0.41
Group B	30/43	67.21 ± 7.89	18.64 ± 7.28	22.67 ± 1.71	0.65 ± 0.42
*t*	0.875	1.094	0.264	2.066	0.291
*P* value	0.346	0.276	0.792	0.040	0.771

D = dioper, IOL = intraocular lens, IOP = intraocular pressure, SD = standard deviation.

There was a statistically significant difference in postoperative SE between the 2 groups (*t* = 2.147, *P* = .033). The preoperative flexion of group A was ‐0.39 ± 0.18 D, and ME was 0.94 ± 0.58 D. There was a statistically significant difference in preoperative expected refraction and postoperative SE between the 2 groups (*t* = 7.738, *P* < .001). In group B, the preoperative expected refraction was ‐0.40 ± 0.20 D and ME was 0.51 ± 0.47 D, and there was no significant difference between the preoperative expected refraction and postoperative SE (*t* = 1.840, *P* = .067). The difference in absolute predicted error between the 2 groups was statistically significant (*t* = 4.929, *P* < .001) (Table 2).

**Table 2:** Visual function index at 3 months (D, mean ± SD).

Groups	Uncorrected vision	After operative IOP (mm Hg)	Best corrected vision	Spherical refraction (D)	Mean absolute refractive error (D)
Group A	0.15 ± 0.14	11.78 ± 6.45	0.05 ± 0.12	1.01 ± 0.94	0.94 ± 0.58
Group B	0.09 ± 0.11	12.34 ± 6.28	0.02 ± 0.09	0.74 ± 0.52	0.51 ± 0.47
*t*	2.879	0.531	1.708	2.147	4.929
*P* value	0.004	0.595	0.089	0.033	0.000

D = dioper, IOP = intraocular pressure, SD = standard deviation.

**Figure 1.**
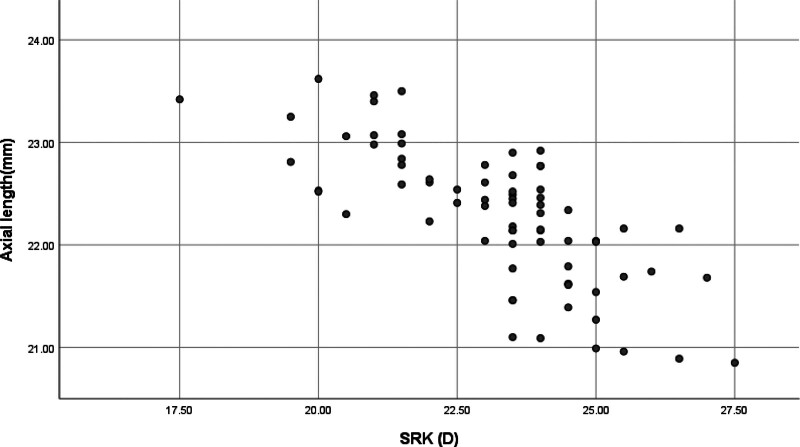
SRK/T formula and eye axis.

**Figure 2.**
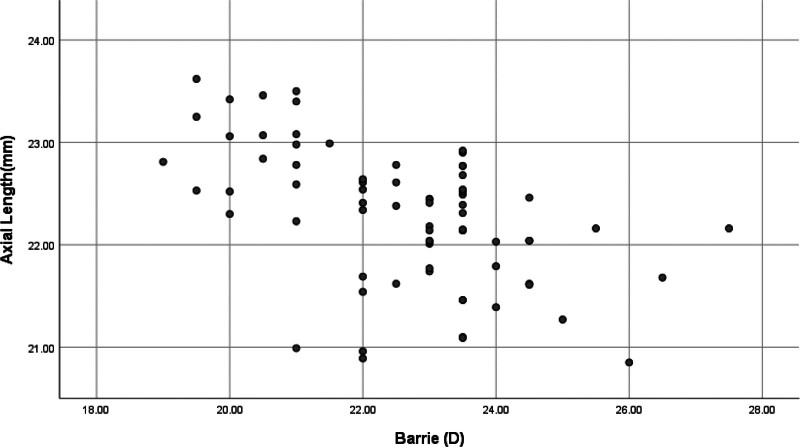
Barrett Universal II and eye axis.

Axis length showed significant negative correlation with ME in both groups (group A: *r* = ‐0.740, *P* < .001; group B: *r* = ‐0.532, *P* < .001) (Figs. 1, 2). There was a significant negative correlation between ME and ACD in both groups (group A: *r* = ‐0.382, *P* = .001; group B: *r* = ‐0.358, *P* = .002), and there was no significant correlation with crystal thickness (group A: *r* = 0.134, *P* = .257; group B: *r* = 0.044, *P* = .710).

## 4. Discussion

The basic anatomic characteristics of primary angle closure are crowded anterior segment and narrow anterior chamber angle. The visible ciliary body processes may indicate anterior segment crowding. In Tekcan H study,^[8]^ the analysis showed significant associations with visible ciliary body processes and axial length, anterior chamber depth, lens thickness, lens vault, and lens-axial length factor. Among these parameters, lens-axial length factor had the highest odds ratio. This may suggest that a relatively large lens is the remarkable factor in anterior segment crowding. Removing the crystalline lens through cataract phacoemulsification can relieve crowding of the angle. In a study by Un Y et al,^[9]^ it was found that, phacoemulsification is equally effective, regardless of whether there is a history of acute attacks, whether it is PAC or primary angle-closure glaucoma, whether it is deep anterior chamber or shallow anterior chamber, whether it is long or short eye.

Phacoemulsification is an important surgical method for treating acute angle-closure glaucoma complicated with cataract in clinical practice. This surgical method not only avoids the complications such as shallow anterior chamber and filtration bubble infection encapsulation caused by traditional trabeculectomy, but also greatly improves the postoperative quality of life of patients.^[10,11]^ However, due to the special characteristics of glaucoma patients’ eye anatomy, it is difficult to accurately judge the diopter of IOL implantation. With the continuous efforts of researchers, there have been 5 generations of formulas for calculating IOL power. The fifth generation IOL power calculation formula is represented by Barrett Universal II and Olsen formula.^[12]^ At present, there is no report on the application of the fifth-generation IOL calculation formula for angle-closure glaucoma patients in China.

Current studies have found that for ACG patients with cataract after filtration surgery, the refractive errors are more myopia than before surgery. It may be due to the relaxation of the suspensory ligament, which causes the position of the IOL to be anterior, as well as the reduction of preoperative IOP and the thickening of the choroid, resulting in the shortening of the axial length and the high diopter of the implanted intraocular lens.^[13–15]^ This research is consistent with previous results.^[16]^In our study, the ME of the 2 formulas were significantly negatively correlated with the ocular axis and anterior chamber depth. It shows that there is still a certain error between the crystal position predicted by the formula and the actual implanted crystal position. In addition, the inherent factors of intraocular lens, such as the A constant, can also affect the calculation results of IOL power calculation formulas. Therefore, our research used the same type of intraocular lens to avoid such errors. It is proved that up to now, the variation of axial length and ACD are still important factors affecting the accuracy of IOL calculation.

Compared with SRK/T formula, the postoperative refractive error of Barrette Universal II formula is significantly smaller. We consider that there may be several reasons as follows: 1. The prediction of IOL strength mainly depends on accurate biometric measurements and the selection of different IOL calculation formulas. With the clinical promotion of various biometric instruments, especially the application of IOL master, the biometric error is getting smaller and smaller.^[16]^ 2. The SRK/T formula is added to the Fyodorov corneal height using A constant value to generate the ACD value to calculate the IOL degree. Barrette Universal II predicted ACD by directly using the individualized anterior chamber depth, axial length and corneal curvature without the need for the Fyodorov corneal height formula.^[17]^ 3. Barrette Universal II formula belongs to the latest generation of formula, which is a thick lens formula based on the paraxial ray tracing technology. Because the formula takes into account the different crystal optical designs corresponding to different crystal degrees, the calculation is more accurate.^[18]^

The study has several limitations. First, there is a lack of controlled trials of different types of IOL, and second, the study was conducted retrospectively. In the next step, a large sample prospective clinical study is needed to confirm the authenticity of the results of this study.

In conclusion, this study shows that phacoemulsification combined with IOL implantation is safe and effective for patients with AGG complicated with cataract, and is worthy of clinical promotion. Compared with the SRK/T formula, we suggest using the Barrett Universal II IOL calculation formula to calculate the postoperative reserved diopter.

## Author contributions

**Conceptualization:** Zhen Ji, Miaomiao Zhang.

**Data curation:** Zhen Ji, Zhonghua Sun, Miaomiao Zhang.

**Formal analysis:** Zhen Ji, Jing Ma, Zhonghua Sun, Miaomiao Zhang.

**Investigation:** Zhen Ji, Jing Ma, Zhonghua Sun.

**Methodology:** Zhen Ji, Zhonghua Sun, Miaomiao Zhang.

**Project administration:** Zhonghua Sun, Miaomiao Zhang.

**Resources:** Miaomiao Zhang.

**Software:** Miaomiao Zhang.

**Supervision:** Miaomiao Zhang.
